# Is vitamin D deficiency in obese youth a risk factor for less weight loss during a weight loss program?

**DOI:** 10.1530/EC-19-0364

**Published:** 2019-10-18

**Authors:** Karolien Van De Maele, Jean De Schepper, Jesse Vanbesien, Monique Van Helvoirt, Ann De Guchtenaere, Inge Gies

**Affiliations:** 1 Division of Pediatric Endocrinology, KidZ Health Castle, UZ Brussel, Vrije Universiteit Brussel, Brussels, Belgium; 2 Zeepreventorium, De Haan, Belgium

**Keywords:** vitamin D, obesity, adolescence, weight loss program

## Abstract

**Background:**

Vitamin D deficiency is common in obese adolescents and a risk factor for insulin resistance. We investigated if prevailing serum 25-OH vitamin D might predict the body fat loss in a group of obese adolescents undergoing a residential weight loss program.

**Methods:**

In 92 (35 male) obese adolescents (aged 10.6–19 years) undergoing a residential weight loss program in Belgium, fasting serum 25-OH vitamin D (25-OH-D), insulin, glucose and lipid levels were measured and body composition was assessed by dual-energy X-ray absorptiometry (DXA).

**Results:**

Baseline median (range) serum 25-OH-D level was 17.7 µg/L (3.8–41.8). In total, 55 adolescents had a serum 25-OH-D below 20 µg/L. In 31 adolescents with a low baseline 25-OH-D level, median increase in serum 25-OH-D was 2.4 µg/L (−4.2 to 7.2) after 10 months. This resulted in normal 25-OH-D levels in seven adolescents, whereas median BMI decreased with 1.0 SDS and body fat percentage diminished with 9.9%. Obese adolescents with or without a 25-OH-D level below or above 20 µg/L at baseline had similar changes in body weight, BMI SDS, body fat percentage and body fat mass at the end of the program. The change in serum 25-OH-D did not correlate with change in serum insulin, BMI SDS or body fat percentage and body fat mass.

**Conclusion:**

Vitamin D deficiency was present in 55 out of 92 obese adolescents at the start of the summer. Serum 25-OH-D concentration did not predict changes in body fat loss after a residential weight loss program.

## Introduction

Obese adolescents are a population known to be at risk for vitamin D deficiency, which is defined as a serum 25-OH-D level below 20 µg/L according to the guidelines set by the Endocrine Society (based on the Institute Of Medicine (IOM) cut-off) (1, 2, 3). Different mechanisms are thought to contribute to the specific vulnerability of the obese adolescent to vitamin D deficiency. Firstly, there might be less exposure to sunlight due to avoidance of outdoor activities and wearing covering clothing (4, 5, 6). Secondly, there is assumed to be a combination of sequestration of the vitamin D in the adipose tissue and volume dilatation of the serum (4, 5, 6, 7, 8, 9). A possible aberrant negative feedback from 1.25-OH_2_-D has also been hypothesized (4, 9). On the other hand, a less efficient 25 hydroxylation of vitamin D has been proposed; however, this hypothesis remains controversial (6, 10, 11, 12).

After cutaneous synthesis and intestinal absorption, vitamin D is hydroxylated twice to produce the active 1.25-OH_2_-D metabolite, which binds to the nuclear vitamin D receptor (VDR) in target cells to stimulate downstream signaling (11). Since both the pancreatic β-cells and the adipocytes express VDR, involvement of vitamin D in glucose homeostasis and fat metabolism is suggested (4, 9, 11, 13). On the one hand, an influence on the glucose homeostasis is hypothesized through less production of insulin by the pancreatic β-cells and decreased peripheral insulin sensitivity by an increased systemic inflammation as 1,25-OH_2_-D is involved in the inhibition of synthesis of Interleukin-6 (IL-6) and tumor necrosis factor-α (TNF-α) (14). On the other hand, a stimulation of lipogenesis, expansion of triglyceride stores and limitation of lipolysis in white fat cells as well increased conversion of pre-adipocytes to adipocytes by compensatory elevated PTH secretion, has been proposed (9, 15).

Some studies show an increase in 25-OH vitamin D level after obtaining weight loss (16, 17). However, the possible causal nature of the correlation between body fat loss and increased serum 25 OH vitamin D level remains controversial. Both obesity and vitamin D deficiency are common in adolescents worldwide, and it is possible that these conditions are coexistent without any cause and effect relationship. Our rationale for thinking there could be an effect of vitamin D3 on weight, was that vitamin D can affect bodyfat status directly by a direct vitamin D receptor-dependent inhibition of the critical molecular components of adipogenesis, as well indirectly by inducing insulin resistance and elevated PTH concentrations. 

Therefore, the main aim of our study is to investigate the predictive value of the vitamin D status in body fat loss in obese adolescents during a residential weight loss program. We hypothesized that adolescents with a low baseline vitamin D level would be at risk for less effective weight loss during the interventional program because of increased insulin resistance.

## Materials and methods

After approval of the Local Ethics Committee of the University Hospital of Brussels, informed consent forms of the parents as well as assent forms from the adolescents were obtained. A total of 92 (35 male) obese (BMI SDS >1.8) adolescents aged between 10.6 and 19 years were prospectively included in July 2012 when entering a 10-month residential weight loss program. A total of 64 adolescents completed the entire program. The explanation for the time delay between data collection (2012–2013) and publication (2019) is that the data was initially collected as part of planned PhD project, which was not completed. The collected data were analyzed for the first time and presented at a conference in 2018 by the participating investigators.

The residential weight loss program combines a nutritional intervention with daily physical activity (18). The diet provides between 1500 and 1800 kcal/day and the physical activities include an individual program (4 h a week) and group activities (regular outdoor activities). Adolescents with chronic diseases (e.g. diabetes mellitus type 1, rheumatoid arthritis) and malabsorption syndromes were excluded as well as adolescents treated with corticosteroids, metformin and vitamin D supplementation.

The studied outcomes combined anthropometric, densitometric and biochemical measurements. Following anthropometric measures were performed at baseline and at the end: body weight and height, waist circumference and body fat assessment by DXA scan. Standing height was measured using a stadiometer with a movable headboard and vertical backboard (SECA, Hamburg, Germany); body weight was measured in light clothing using a class 3 SECA weighing scale. The total body fat percentage was assessed using a DXA scan (Lunar, GE Healthcare). BMI was calculated from measured body weight and body height and expressed as SDS according to the Flemish growth charts (2014 data). Pubertal development was assessed according to Tanner stages.

A blood sample was taken by a venipuncture after an overnight fasting at baseline and repeated at the end of the program in May 2013 in case of 25-OH vitamin D <20 µg/L at baseline. Following biochemical parameters were determined using standard laboratory techniques: glucose, insulin, total cholesterol, LDL cholesterol, HDL cholesterol, triglycerides and 25-OHD level. Serum 25-OH vitamin D was measured by electrochemiluminescence (Cobas 8000/Roche Diagnostics). The metabolic syndrome was defined by the IDF standards (based on waist circumference, blood pressure, triglycerides, HDL cholesterol and glucose level) (19).

The adolescents were categorized in different obesity stages according to the Flemish Growth charts (20): overweight is a BMI SDS between 1.3 and 2.3, stage 1 obesity is a BMI SDS between 2.3 and 3.0, stage 2 obesity is a BMI SDS between 3.0 and 3.5 and stage 3 obesity is a BMI SDS above 3.5. Vitamin D deficiency was defined as a 25-OH-D level below 20 µg/L.

All statistical analysis were performed using SPSS, version 25. Paired *t*-tests for the comparison of repeated-measures and Pearson correlation analysis were performed, while Mann–Whitney *U* test was used to compare the anthropometric changes between children with a 25-OH vitamin D concentration below or above 20 µg/L. *P* values <0.05 were considered statistically significant.

## Results

### Baseline characteristics and comparison between adolescents with a normal or deficient vitamin D status

The baseline anthropometric characteristics of the 92 adolescents at start of the program are summarized in Table 1. The vast majority of the adolescents were Caucasian (*n* = 85); other ethnic groups were African (*n* = 6) and Latin (*n* = 1) adolescents. Three female adolescents were at Tanner breast stage 2, 10 at stage 3 and 44 at stages 4 and 5. Eleven male adolescents were at Tanner genital 2, 2 at stage 3 and 22 at stages 4 and 5. The 28 adolescents leaving the program prematurely were significantly older (15.8 vs 14.4 years), smaller (height SD −0.16 vs 0.33) and had a higher fat percentage (51.55 vs 50.40%) than those who completed the 10-month program, probably because of different gender distribution (8/28 males versus 27/64 males).

**Table 1:** Comparison of anthropometric and biochemical characteristics between obese adolescents leaving and completing the weight loss program.

	Total group (*n* = 92)	Program not completed (*n* = 28)	Program completed (*n* = 64)	*P* value
Male/female	35/57	8/20	27/37	0.22
Age (years)	14.7 (10.6–19.0)	15.8 (11.9–19.0)	14.45 (10.6–18.7)	**0.002**
Weight SDS	2.75 (1.52–4.41)	2.79 (1.52–4.18)	2.75 (1.59–4.41)	0.83
Height SDS	0.26 (−2.48 to 2.84)	−0.16 (−2.48 to 1.59)	0.33 (−1.93 to 2.84)	**0.01**
BMI SDS	2.74 (1.82–4.00)	2.81 (1.96–4.00)	2.62 (1.82–3.91)	0.13
Body Fat (%)	50.80 (37.50–59.30)	51.55 (38.4–56.3)	50.40 (37.5–59.3)	**0.05**

Values are expressed as median with the range in paracentesis. Bold indicates statistical significance, *P* < 0.05.

**Figure 1:**
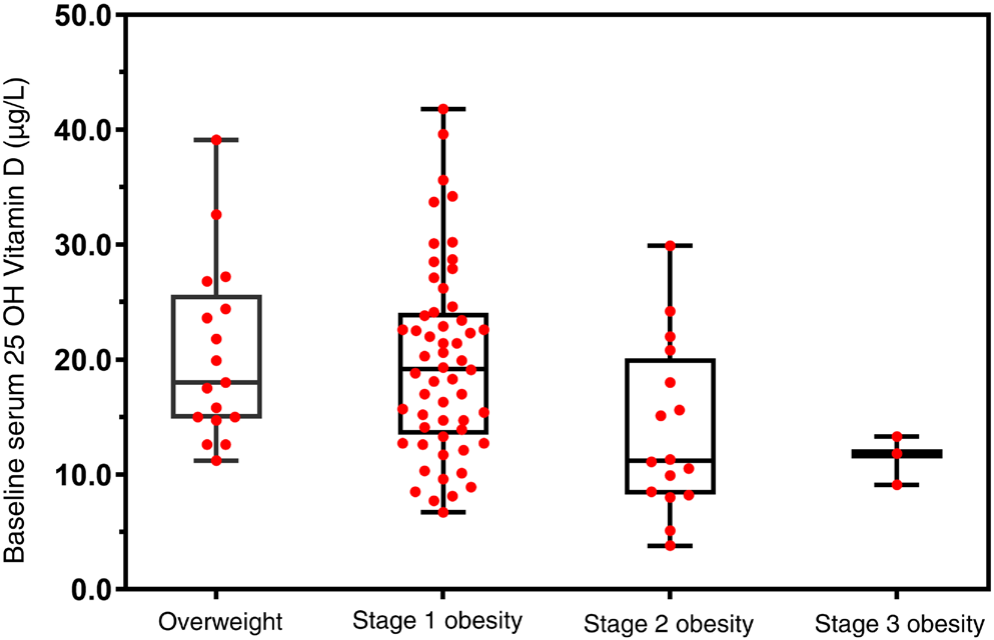
Baseline serum 25-OH-D levels according to different obesity stages.

Baseline median (range) serum 25-OH-D level was 17.7 µg/L (3.8–41.8). In total 55 adolescents had a serum 25-OH-D below 20 µg/L. There was no effect of age, gender or pubertal status difference on serum 25-OH vitamin D. Baseline serum 25-OH vitamin D was inversely correlated with the weight SDS (*r* = −0.302; *P* = 0.003) and BMI SDS (*r* = −0.305; *P* = 0.003), but not with body fat percentage (*r* = 0.043; *P* = 0.686). When patients were categorized according the different obesity stages, there was no significant difference (*P* = 0.092) in 25-OH vitamin D levels (Fig. 1). Body fat percentage ranged between 37.5 and 59.3% (median 50.8%). Females had a higher body fat percentage than boys (51.5 vs 48.7%, *P* = 0.005).

Serum fasting insulin ranged between 36 and 294 pmol/L, while fasting hyperinsulinemia (>115 pmol/L based on the used assay (21)) was present in 43 adolescents. The metabolic syndrome defined by the IDF standards was present in 47 obese adolescents. Baseline median (range) lipid levels were respectively 129 mg/dL total cholesterol (75–209), 74 mg/dL LDL cholesterol (29–153), 37 mg/dL HDL cholesterol (21–72) and 74 mg/dL triglycerides (36–189). Dyslipidemia was present in 60 adolescents, defined as at least one of the following: LDL cholesterol above 130 mg/dL, HDL cholesterol below 40 mg/dL or triglycerides above 130 mg/dL.

Obese adolescents with a low 25-OH-D at the start had similar baseline serum insulin concentrations (110 vs 99 pmol/L), body fat percentage (50.9 vs 50.7%), body fat mass (49.4 vs 47.9 kg) and lipid profile (data not shown), than those with a normal vitamin D status, as shown in Table 2.

**Table 2:** Comparison of groups according to baseline 25-OH-D level above or below 20 µg/L.

	Baseline 25-OH-D <20 µg/L (*n* = 36)	Baseline 25-OH-D >20 µg/L (*n* = 29)	*P* value
Baseline serum insulin (pmol/L)	110 (37–247)	99 (36–294)	0.52
Baseline body fat percentage (%)	50.4 (40.2–59.3)	50.7 (37.5–55.6)	1.0
Baseline body fat mass (kg)	47.8 (29.2–88.7)	47.4 (26.2–83.5)	0.61
Baseline total cholesterol (mg/dL)	127 (75–199)	130 (76–202)	0.61
Baseline LDL cholesterol (mg/dL)	73 (39–117)	77 (29–153)	0.47
Baseline HDL cholesterol (mg/dL)	38 (28–59)	35 (28–54)	0.11
Baseline triglycerides (mg/dL)	78 (42–128)	67 (40–128)	0.61
Change in weight (kg)	−24.4 (−52.1 to −8.2)	−23.5 (−55.2 to −8.2)	0.61
Change in BMI SDS	−1.09 (−2.33 to 0.5)	−1.32 (−2.32 to −0.02)	0.13
Change in body fat percentage	−11.25 (−38.2 to 0.5)	−14.9 (−30.9 to −2.6)	0.10

Data are expressed as median and range.

### Changes in body composition and serum 25-OH vitamin D levels

**Figure 2:**
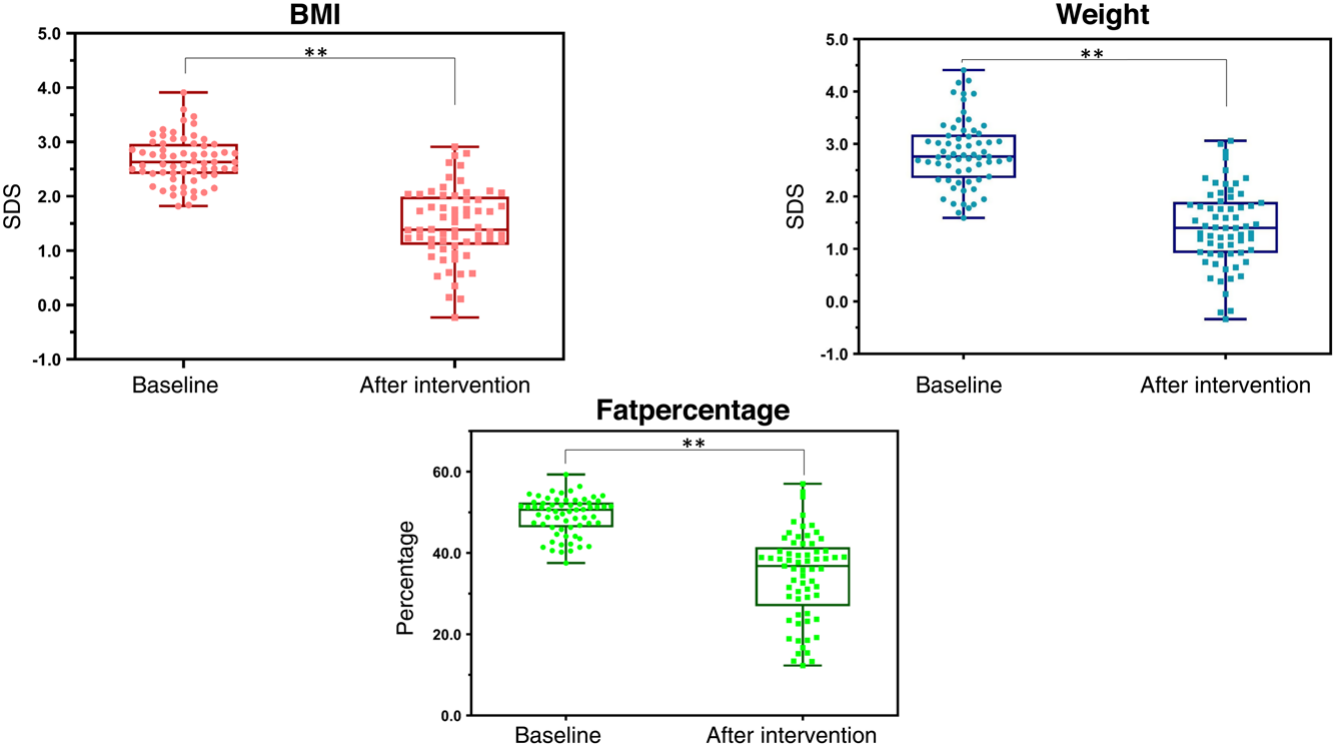
Comparison of anthropometric measurements before and after the intervention. Changes in weight SDS (blue), BMI SDS (red) and body fat percentage (green) after the 10-month intervention. Whiskers express range from minimum to maximum. Overall significance ** is *P* < 0.001.

Median decrease in body weight, BMI SDS and body fat percentage after 10 months (*n* = 64) were respectively −24.0 kg (−55.2 to +3.4); −1.1 SDS (−2.3 to 0) and −12.3% (−38.2 to 0.5) (Fig. 2).

In 31 adolescents with a low baseline 25-OH-D (<20 µg/L) and repeated measurements, median serum 25-OH-D, measured in May, was 16.3 µg/L (6.7–26.6), resulting in a median increase of 2.4 µg/L (−4.2 to 7.2) (*P* < 0.001) and a normalization of 25-OH-D levels in seven adolescents.

The change in serum 25-OH-D was not related with the changes in serum insulin (*r* = −0.074; *P* = 0.82), BMI SDS (*r* = −0.332; *P* = 0.068) or body fat percentage (*r* = −0.349; *P* = 0.054). Obese adolescents with a low 25-OH-D at the start had similar change in weight (−24.4 vs −23.5 kg), BMI SDS (−1.1 vs −1.3 SDS) and body fat percentage (−11.2 vs −14.9%) after the intervention than those with a normal vitamin D status, as shown in Table 2.

## Discussion

Vitamin D deficiency was found in more than half of obese adolescents at the beginning of a residential weight loss program, which resulted in a significant loss in body fat percentage with 12.3% and was associated with a median increase of circulating 25-OH-D with 2.4 µg/L. Less than a quarter of the adolescents with a vitamin D deficiency at the start of the program had a normal vitamin D status at the end of the intervention. Against our expectations, neither baseline serum 25-OH-D nor the change in 25-OH-D predicted the weight loss outcomes at the end of the program.

Previous studies have identified the obese population being especially at risk for vitamin D deficiency. Studies regarding the prevalence in obese adolescents are sparser than in adults, but still report a high prevalence of vitamin D deficiency, ranging from 34 to 100%, regardless of the age of the adolescents (2, 3, 7, 8, 11, 15). Serum 25-OH vitamin D levels however depend greatly on the season in North and West European countries, with the highest level during the summer time. We found that in the month of June, 55 out of a group of 92 Belgian obese adolescents were vitamin D deficient. We do expect much lower 25-OH-D levels during winter time, as we previously observed the highest serum 25-OH-D levels from June to September in a Belgian population of children and adolescents with cystic fibrosis (22).

Since the adolescents in our residential program received a healthy diet and did frequent outdoor activities, we expected their vitamin D intake and cutaneous vitamin D production to be higher at the end of the program. We only observed a slight increase of 2.4 µg/L in those adolescents with a low baseline 25-OH-D level. In some studies, weight loss in obese adolescents was found to result in a subsequent increase of the 25-OH vitamin D level ranging from 1.8 to maximum 5 µg/L (16, 17).

Since a vitamin D deficit correlates with insulin resistance, we suspected that the low vitamin D status at start would play a counteractive role in the weight loss process (7). We however did not observe a lower weight loss or fat mass loss in those adolescents with a vitamin D deficiency. Nevertheless, the vitamin D-deficient children in our study had comparable serum insulin levels at start of the program, suggesting a comparable degree of insulin sensitivity in the adolescents both with 25-OH vitamin D levels above and below 20 µg/L.

There is still uncertainty whether there is an actual causal relationship between the vitamin D and effective weight loss. Studies based on bi-directional Mendelian randomization analysis argue that vitamin D deficiency is rather a cause of the obesity than an actual modulator of the weight loss process (23). Since the serum 25-OH-D changes did not correlate with any changes in body composition nor could be used to predict the weight outcomes at the end of the program, the actual clinical implication of low vitamin D levels seem limited for now, based on the current results.

The most important strength of the study is the unique study population, resulting from the standardized residential multidisciplinary weight loss intervention. In terms of standardization, the residential program is superior to outpatient regimens, where the amount of physical activity and caloric intake are more difficult to compare. In our study, we know the adolescents had a similar dietary supply of vitamin D and exposure to sunlight. Since the serum levels were measured twice during the same season, we additionally limited the known seasonal variability in the serum levels of 25 OH vitamin D. In addition, not only body weight but also whole body fat percentage, using well-standardized measurement by DXA, was assessed in our study.

The key limitation of our study is the fact that we only have a second serum vitamin D measurement of a subgroup of adolescents who already started the intervention with a value below cut-off. We can only speculate whether a higher baseline value would result in a more pronounced increase in vitamin D levels after the intervention. The significant dropout rate reported in our study is due to the residential program offered at the Zeepreventorium, in which adolescents are required to leave their home-environment for a complete school year, putting both physical stress (coping with daily physical exercises) and psychological stress (leaving the home-environment) on the participants.

Future work should focus on the identification of the factors contributing to a low vitamin D status in obese adolescents as neither age nor body fat percentage are involved, suggesting a potential important role for low sunshine exposure in this particular population.

## Conclusion

Vitamin D deficiency was found in more than half of the obese adolescents starting a residential, standardized weight loss program. A slight increase of serum 25-OH-D was observed at the end in those adolescents who had a vitamin D deficiency at the start, resulting in a persistent vitamin D deficiency in 77.4%. Obese adolescents with or without a vitamin D deficiency at start of the weight loss program had similar body fat loss, questioning the need for a systematic vitamin D supplementation, at least for increasing weight loss.

## Declaration of interest

The authors declare that there is no conflict of interest that could be perceived as prejudicing the impartiality of the research reported.

## Funding

This research did not receive any specific grant from any funding agency in the public, commercial or not-for-profit sector.

## Statement of ethics

This study was conducted according to the guidelines laid down in the Declaration of Helsinki and all the procedures were approved by the Ethics Committee of the University Hospital of Brussels. An assent of the adolescents included in this study and a written informed consent of the parents were obtained.

## Author contribution statement

I G, J D S and M V H designed the study and performed the measurements. I G, J D S and K V D M wrote the paper with important input from all other authors. All authors performed a critical revision of the final manuscript and agreed with the final content.
